# Effect of Plyometric Training on Speed and Change of Direction Ability in Elite Field Hockey Players

**DOI:** 10.3390/sports6040144

**Published:** 2018-11-12

**Authors:** Jasdev Singh, Brendyn B. Appleby, Andrew P. Lavender

**Affiliations:** 1 School of Physiotherapy and Exercise Science, Curtin University, Perth 6102, Australia; jasdev.singh1@graduate.curtin.edu.au; 2 Hockey Australia High Performance Unit, Perth 6102, Australia; brendyn.appleby@hockey.org.au; 3 School of Medical and Health Science, Edith Cowan University, Perth 6027, Australia

**Keywords:** change of direction, speed, plyometric, drop jump, eccentric, team sport, hockey

## Abstract

This study investigated the effects of two plyometric training protocols on sprint and change of direction (COD) performance in elite hockey players. A parallel-group randomized controlled trial design was used and seventeen elite male and female field hockey players were randomly allocated into either low-to-high (L-H, *n* = 8) or high-to-low (H-L, *n* = 9) training groups. Each group performed separate variations of the drop jump exercise twice weekly for six weeks, with an emphasis on either jump height (L-H) or drop height (H-L). Performance variables assessed included sprint times over 10 m and 20 m, as well as 505 time. A two-way repeated measures analysis of variance was performed and Cohen’s d effect sizes (ESs) were calculated. The H-L group displayed a significant small ES improvement from baseline to post-training in the 10 m sprint (1.893 ± 0.08 s pre vs. 1.851 ± 0.06 s post) (ES = −0.44) (*p* < 0.05). Differences between groups for 10 m and 20 m sprint performance failed to reach statistical significance, and no significant differences were observed within or between groups for 505 time. These findings highlight the difficulty in substantially enhancing speed and COD ability in highly trained athletic populations through the addition of a low volume, short duration plyometric training protocol.

## 1. Introduction

Change of direction (COD) ability refers to an athlete’s ability to rapidly decelerate, reverse, or change direction of movement, and accelerate again in a new direction [1]. Field hockey (as distinguished from ice hockey, herein referred to simply as hockey) is a sport that requires frequent changes of direction and repeated sprint efforts throughout the duration of a match [2]. Hockey players, as in many other team sports such as soccer, rugby, and Australian Rules football, depend heavily on their speed and COD ability when attempting to evade opposition defenders, closely mark opposition attackers, or gain positional advantage [1,2]. Whilst perceptual abilities such as knowledge of situations and anticipation also contribute to an athlete’s overall sport-specific agility, COD is the term used to address the physical aspects of agility, without consideration of the cognitive and decision-making factors [3]. Three main phases exist within the performance of a COD task: the braking (eccentric) phase, plant (isometric) phase, and propulsive (concentric) phase [4]. Strength and conditioning (S&C) practitioners often implement a variety of training methods aimed at developing the key physical qualities that influence speed and COD ability. The ability to produce powerful concentric muscle actions in order to maximize propulsive force is believed to be a key determinant of speed [5,6], whilst COD ability is believed to also be dependent on eccentric and isometric strength of the lower limb muscles, as well as neuromuscular control throughout the various phases of a COD task [4,7,8].

Traditionally, commonly used methods of enhancing these physical qualities include strength and power training exercises such as squats and deadlifts, as well as power cleans and other weightlifting variants [9,10]. Another popular means of improving speed and COD ability is through plyometric training, which refers to a wide range of jumping, hopping, and bounding exercises designed to utilize the elastic nature of the stretch-shortening cycle (SSC) in order to produce greater forces than would normally be produced by concentric-only muscle actions [9,11]. Previous studies have demonstrated that enhancements in speed and COD ability can be achieved through the use of plyometric training methods [9,12,13,14,15], however, the majority of this research has focused on the development of concentric power of the lower limbs and resulting enhancements in performance during the propulsive phase of COD tasks. A relative lack of studies has investigated the most appropriate training methods for the development of the physical qualities that influence performance during the braking and plant phases of COD tasks, despite the established importance of deceleration to overall COD ability [16,17].

Some more recent studies, however, have demonstrated that improvements in muscle power, speed, eccentric and isometric strength, and various COD and jumping performance tests can be achieved through the use of training interventions involving eccentric overload [18,19,20,21]. Lockie and colleagues [22] found that emphasizing deceleration during speed and agility training improved unilateral strength, as well as performance in COD tests and 40 m sprint time. It has been suggested that eccentric overload training resulting in enhanced eccentric strength can improve athletes’ COD performance during the braking and plant phases, as athletes who are able to produce greater force eccentrically are able to produce greater decelerations from faster approach velocities [20,23]. In support of this, de Hoyo and colleagues [24] found that ten weeks of eccentric overload training resulted in improved kinetic parameters including time spent braking, relative peak braking, and relative braking impulse during COD tasks. Further, increased braking impulse may enable more rapid reacceleration during a COD task as a result of the storage and utilization of elastic energy [1]. However, Bourgeois and colleagues [18] found that although eccentric overload training resulted in greater isometric strength and improvements in 180° COD performance, performance in a 45° COD task improved only in athletes categorized as fast, and worsened in slower athletes. The authors suggested that performance benefits from eccentric overload training may be specific to the COD task (such as the angle of COD and the distance of sprints between changes of direction) and athlete category (such as faster or slower). Nonetheless, the results of these studies are promising with regards to the enhancements in sprint and COD performance that can be achieved through eccentric overload training.

The importance of speed and COD ability to team sport athletes is well established [2,4,9,10], and as such, S&C practitioners are constantly seeking information regarding the most effective training methods for the development of these athletic qualities. Although many training methods have been found to be successful at enhancing sprint and COD performance, there appears to be little agreement regarding which methods of training are optimal. This may be in part because, as mentioned above, enhancements in COD ability may be dependent on the specificity of the training intervention to the specific COD task, as well as the type and level of athlete involved [18]. Plyometric exercises have been found to be beneficial and are commonly implemented in speed and COD training, however, the focus of these exercises has primarily been on the development of concentric power and resulting enhancements during the propulsive phase of sprint and COD tasks [9,12,13,14,15]. Given the recent interest and promising enhancements observed in sprint and COD performance following training interventions involving eccentric overload, it is possible that plyometric exercises slightly altered to include an accentuated eccentric phase may have a beneficial effect on speed and COD ability. Additionally, very little research has investigated the effects of plyometric training in hockey players specifically, and no studies to date have investigated the effects of plyometric training on speed and COD ability in hockey players at the elite level.

The present study aimed to investigate the effects of two short-term plyometric training protocols on speed and COD ability in international level hockey players. Specifically, the authors aimed to examine the effects of a plyometric training protocol performed with an accentuated eccentric portion of the exercise, compared with a conventional plyometric training protocol, on sprint and 180° COD performance. We hypothesized that whilst both interventions would enhance sprint performance, the training group with a novel accentuated eccentric portion of the exercise would display greater improvements in COD performance compared with the conventional plyometric training group, due to improved performance in the braking and plant phases.

## 2. Materials and Methods

### 2.1. Participants

**Figure 1:**
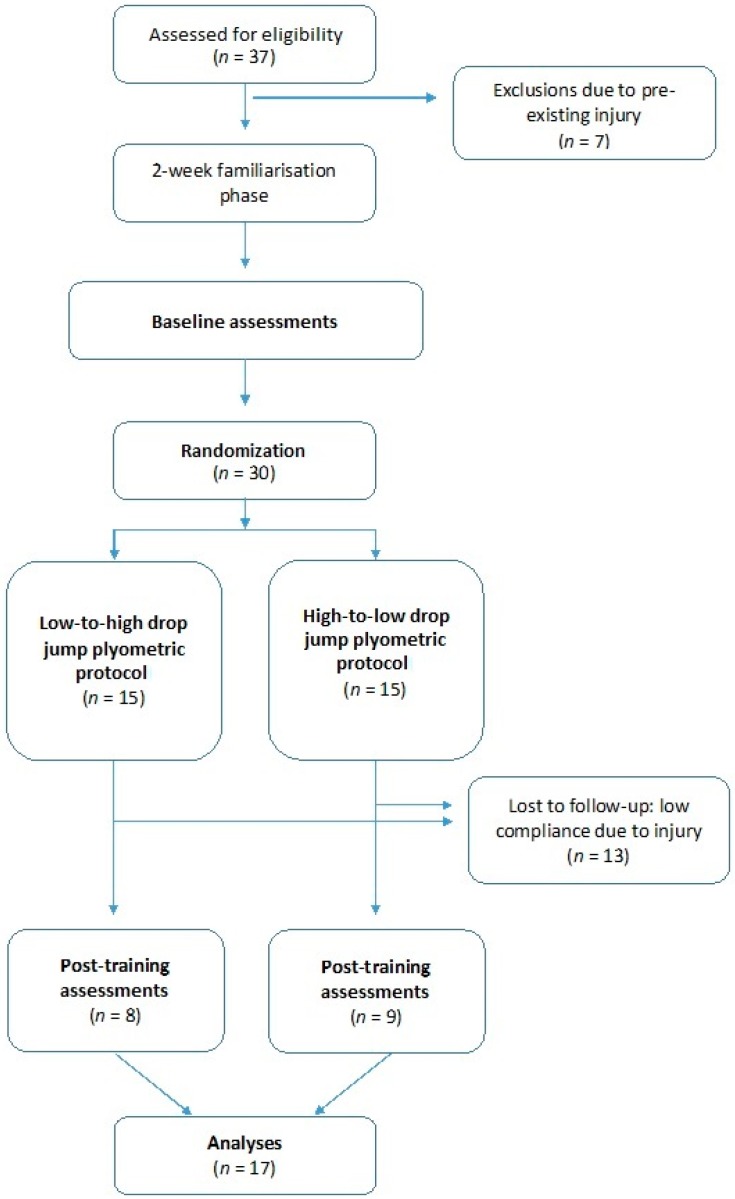
Study design and sample size at various stages of the study.

Thirty-seven elite athletes from the men’s (*n* = 16) and women’s (*n* = 14) Hockey Australia High Performance Program were invited to participate in this study. All national squad field players were eligible for inclusion except in the case of pre-existing injuries, which resulted in the exclusion of seven participants prior to baseline. As a result of individual subject modifications due to overall training load and physical management concerns, a further thirteen participants failed to achieve minimum compliance of plyometric training set at >70% [25] (Figure 1). The high rates of drop-out due to injury during the intervention period were not related to the study protocol itself, but rather occurred during the performance of additional skills training and games completed as part of the participants’ regular national squad training. Age, height, body mass, and number of international caps for the final sample (males, *n* = 11; females, *n* = 6) were 23 ± 2.4 years, 1.76 ± 0.76 m, 73 ± 8.5 kg, and 45 ± 43 caps (mean ± SD). All participants received written and verbal information about the interventions and their role in the study and gave their informed consent for inclusion before they participated in the study. The study was conducted in accordance with the Declaration of Helsinki, and the protocol was approved by the Curtin University Human Research Ethics Committee (HRE2017-0107).

### 2.2. Procedures

A two-group, parallel, randomized controlled trial design was used for this study. As part of their High Performance Program participants were completing five to six additional training sessions a week throughout the study period, comprising of a combination of skills, conditioning, and strength training. The testing and training procedures involved in this study were integrated into the High Performance Program schedule. All eligible participants who agreed to take part in the study and provided written informed consent were randomized into low-to-high (L-H) or high-to-low (H-L) drop jump training groups. Randomization was generated electronically following the completion of baseline testing.

### 2.3. Assessments

**Figure 2:**
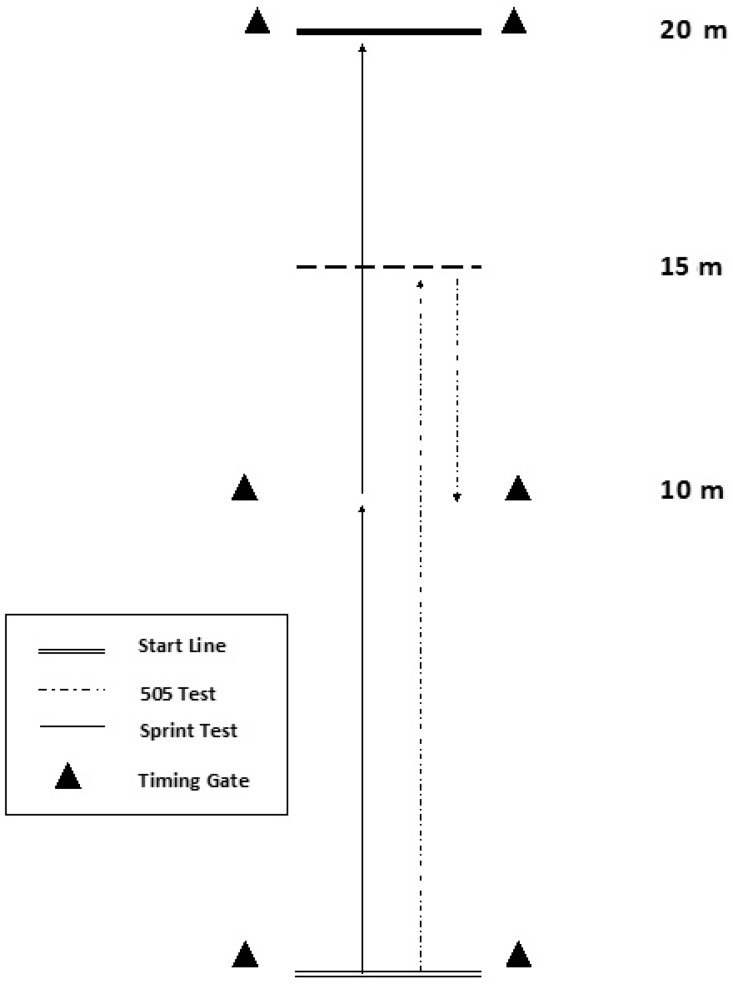
Structure and dimensions of the sprint and change of direction (COD) tests.

All assessments related to the study were conducted at the Western Australian Institute of Sport, Perth, Australia. Baseline and post-training assessments were conducted in the week immediately prior and the week immediately following the training intervention, respectively. Assessments were scheduled before any training sessions on that day in order to minimize the negative influence of fatigue on performance. Assessments were performed on an indoor synthetic track and involved a 20 m sprint (with 0–10 m split) and 505 COD test, which followed previously reported procedures [26]. The test area set-up and positioning of the timing gates (SMARTSPEED Pro, Fusion Sport Pty Ltd., Brisbane, Australia) are illustrated in Figure 2. An adequate warm up was performed by each participant 15 min prior to performance assessment, consisting of three minutes of jump rope skipping, followed by eight minutes of various lower body dynamic stretches, and finally speed runs at self-determined gradually increasing intensities. Each participant then completed two trials of the 20 m sprint and four trials of the 505 COD test, turning off each leg twice. Sufficient rest of at least three minutes was allowed between trials to minimize the influence of fatigue and ensure each trial was performed at maximum intensity. The order of assessments was randomized across participants and sessions. Measures of time for 0–10 m and 0–20 m splits were recorded for the sprint assessment, and 505 time was recorded as a measure of COD ability. All times were recorded to the nearest 0.001 s.

### 2.4. Training Protocol

A two-week familiarization phase was included in the training protocol prior to randomization and baseline testing, during which time all participants performed two sets of four reps of each of the L-H and H-L drop jumps twice weekly. Participants also practiced the 505 test once each week during this phase, with the purpose of controlling for the effect of motor learning on improvements in COD and sprint performance [25].

**Figure 3:**
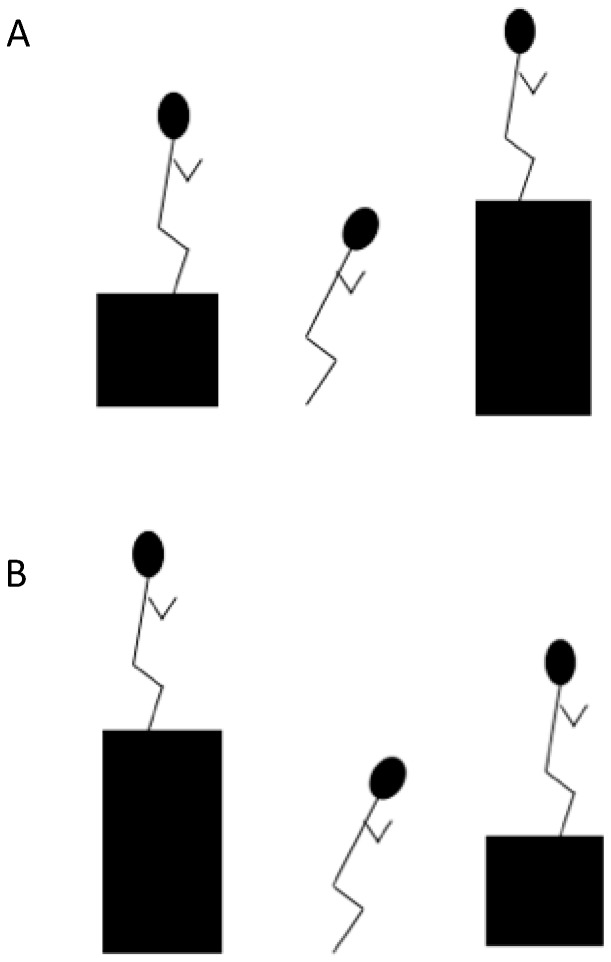
Illustration of the Low-to-High (L-H) drop jump (**A**), emphasizing jump height, and High-to-Low (H-L) drop jump (**B**), emphasizing drop height. Boxes were placed 100 cm apart.

A standardized warm up consisting of 15 min of aerobic exercise, general mobilization, and ballistic exercises was completed by all participants before each training session throughout the intervention period. The plyometric drop jumps performed by the two training groups are illustrated in Figure 3. The intention was to emphasize drop height in the H-L training group, placing a greater demand on the eccentric portion of the exercise [27,28,29]. The L-H training group performed conventional drop jumps with an emphasis on vertical jump height. Both training groups completed five sets of four reps of their respective drop jump exercises twice weekly for a duration of six weeks, with 48–72-h rest periods allowed between training sessions. Due to the high intensity nature of the plyometric exercises performed, and as the exercises were an addition to the athletes’ regular training schedule, a relatively low volume protocol was required. In order to achieve overload participants were progressed individually, based on analysis of drop jump execution and technique by accredited S&C coaches (Australian Strength and Conditioning Association, Pro-structure). For the L-H group, drop height was fixed at 30 cm for women and 40 cm for men, based on recommendations of previous research [27,28,29]. Jump height was progressed by an increase in height of the finishing box. Mean finishing box height for the L-H group progressed from 75 cm in the first week to 90 cm in the final week of the intervention. Conversely, progression for the H-L group involved an increase in drop height, as determined by the height of the starting box, with the finishing box height fixed at 30 cm for women and 40 cm for men. Mean starting box height for the H-L group progressed from 70 cm in the first week to 85 cm in the final week of the intervention. The plyometric protocol was integrated into each athlete’s regular strength training program. These programs involved the completion of four to six sets of three to eight repetitions of various lower and upper body resistance training exercises such as squats, deadlifts, step-ups, bench press, and pull-ups. All athletes across both groups performed these strength training exercises at the same relative intensities.

### 2.5. Statistical Analyses

Statistical analyses were conducted using IBM Statistics for Windows, version 24.0 (IBM Corp., Armonk, NY, USA). The best times at baseline and post-training for all assessments (fastest trial on preferred leg for the 505) were used for analyses. All baseline and post-training data for the 10 m, 20 m, and 505 COD tests were assessed for normality using a Shapiro-Wilk test. A two-way repeated measures analysis of variance was then conducted with one between-subjects factor (group: H-L vs. L-H) and one within-subjects factor (time: baseline vs. post-training). Where a significant *F*-value occurred for effects of group, time, or the group-time interaction, Fisher’s Least Significant Difference (LSD) post-hoc procedures were performed. The level of significance was set to *p* < 0.05 and all values are reported as mean ± standard deviation. Additionally, Cohen’s d effect sizes (ESs) were determined using a custom spreadsheet [30] and are expressed with 90% confidence limits. For comparison of group means, outcomes were adjusted to the fastest baseline value. Where the confidence interval overlapped thresholds for substantial positive and negative values, the effect was deemed unclear [31]. The effect was otherwise clear and reported as the magnitude of the observed value. Threshold values for assessing magnitudes of ES were 0.20, 0.60, 1.2, and 2.0 for small, moderate, large, and very large, respectively [31].

## 3. Results

**Figure 4:**
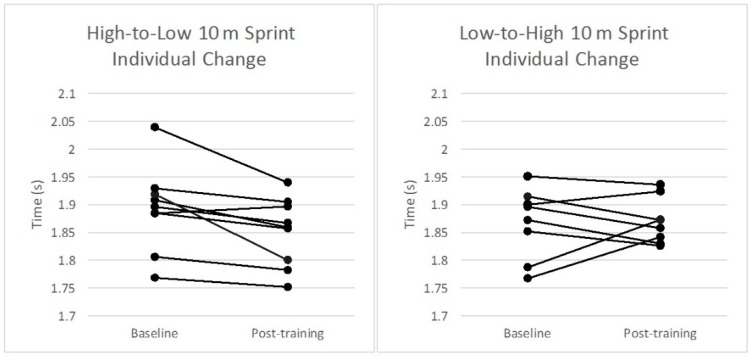
Individual changes in performance in the 10 m sprint from baseline to post-training for each group.

**Figure 5:**
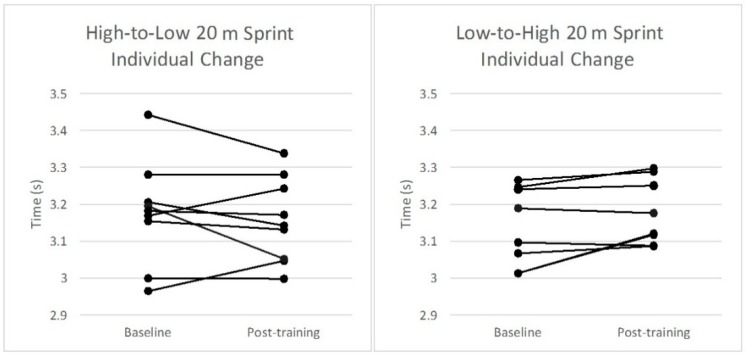
Individual changes in performance in the 20 m sprint from baseline to post-training for each group.

**Figure 6:**
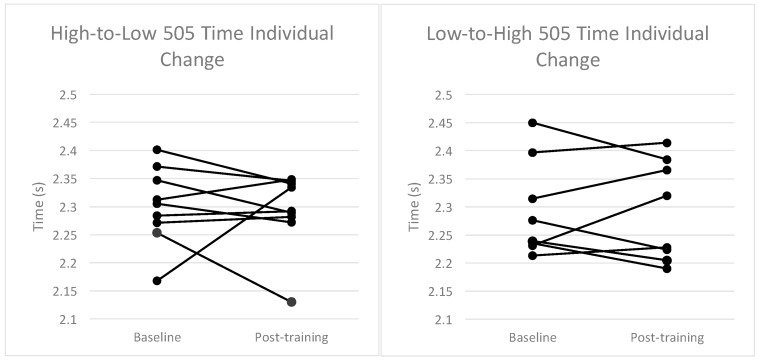
Individual changes in performance in the 505 test from baseline to post-training for each group.

The changes in group means for the H-L and L-H groups from baseline to post-training and ESs for each performance variable assessed are displayed in Table 1. A small significant within-subjects effect for the group–time interaction was found in 10 m sprint time (F_1,7_ = 7.1, *p* < 0.05) from baseline to post-training in the H-L group (Table 1). Differences observed between groups for 10 m and 20 m sprint times failed to reach statistical significance (ES = 0.57 and 0.40, respectively). No statistically significant differences were observed within or between groups for 505 time. Figure 4, Figure 5 and Figure 6 illustrate the individual changes in performance from baseline to post-training for each performance variable.

The results show similar performance at baseline and post-test measures as well as changes from pre- to post-intervention between the groups across 10 m (Figure 4), 20 m (Figure 5), and 505 tests (Figure 6) (Table 1).

## 4. Discussion

The aim of the present study was to examine the effects of two plyometric training protocols, performed in either a conventional manner or with an accentuated eccentric phase of the exercise, on measures of sprint and COD performance in elite hockey players. Some improvement in 10 m sprint performance was observed in the H-L group following the training intervention. While this result is interesting, the results should be interpreted with some caution. Since the number of participants in the study is low, a large difference by one participant may have a profound effect on the overall group score. It is important to note that the slowest participant in the H-L group improved the most while the two fastest in the L-H group got slower, as did a third participant. The difference in times are approximately 50 to 100 milliseconds, so these are very slight changes and we should be careful when drawing conclusions from these results. No other significant improvements in performance were observed for either group. These findings highlight the difficulty in practical interventions designed to substantially enhance speed and COD ability in highly trained athletic populations through the addition of a low volume, short duration plyometric training protocol.

Whilst little research has investigated the effects of drop jumps performed in the manner of the H-L group, much more research has involved conventional style drop jumps, such as those performed by the L-H group, and found mostly positive effects on measures of lower limb muscular power and linear sprint performance [12,15,32,33,34]. It has previously been posited that the rate of progress in strength and power development diminishes with increased strength levels and resistance training experience, and as a result, a limited scope exists for gains in muscular strength and power and their related performance variables in elite athletes or athletes with greater resistance-training experience [35,36]. Additionally, strength- and power-related training performed concurrently with high volumes of running can significantly decrease gains in lower body strength- and power-related measures [37]. Ronnestad and colleagues [38] found that the addition of a specific plyometric training program in professional soccer players who were already performing high volumes of strength and sport-specific training during their preseason preparation phase produced no further improvements in strength- and power-related measures. It is therefore possible that the high training experience of the elite population of athletes used in this study, together with the high volumes of additional strength, conditioning, and sport-specific training concurrently performed throughout the intervention period, contributed to the general lack of significant improvements observed. Furthermore, due to the integration of the training intervention into an ongoing high performance training program, and the intensive nature of the drop jumps performed, this intervention was of relatively low volume and short duration, which may have also contributed to the lack of significant improvements in performance. Future research in elite field sport athletes may involve higher volumes of plyometric training than utilized in the current study, however, risk of overload injury in athletes with an already high training load may be a concern. However, constraints on program duration and available training volume in team sport athletes are common in real world scenarios, and as such, these findings are of practical relevance. Practitioners should be aware of the seemingly limited scope for substantial improvement in sprint and COD performance in highly trained athletic populations in order to set appropriate short-term physical performance goals. Available duration and training volume, as well as the type and volumes of additional training concurrently being performed, are possible constraints that should be taken into consideration.

Given the limited scope for improvement among highly trained athletes, the large volumes of additional training concurrently being performed, and the relatively low volume and short duration of the intervention discussed above, the significant enhancement in 10 m sprint performance observed in the H-L group provides promise for this type of training as a means of enhancing short distance sprint performance, and warrants further investigation. Previous biomechanical analyses have demonstrated that incremental increases in drop height during drop jump performance are associated with increased vertical ground reaction force, ground contact time, braking and total impulse, and greater activation of the rectus femoris muscle, which acts eccentrically to decelerate the body upon landing [27,28,29]. The H-L drop jump training protocol implemented in the present study was designed based on these previous findings, such that the H-L training group would experience greater loading during the eccentric portion of the exercise compared with the L-H group. Recent studies have demonstrated that eccentric overload and eccentrically-accentuated training can enhance muscle strength, power, and SSC function, resulting in improved sprint and COD performance [18,19,21,39,40]. In their systematic review of the chronic adaptations to eccentric training, Douglas and colleagues [39] highlighted the growing body of evidence in support of eccentric training as a means of inducing a novel adaptive signal for neuromuscular adaptations that can improve athletic qualities such as those mentioned above, including strength, power, and SSC performance. The review explored the mechanisms likely underlying the observed improvements in muscle power, and SSC performance, including an increased ability to rapidly recruit large motor units, enhanced eccentric force control and coordination during the eccentric phase within SSC tasks, and increases in tendon stiffness and cross-sectional area, which influence the storage and return of elastic strain energy. Whilst the research surrounding eccentrically-biased training has primarily revolved around strength training methods so far, the findings of the current study warrant further investigation into the use of eccentrically-accentuated plyometric exercises for the enhancement of athletic qualities. It is possible that plyometric exercises performed with an accentuated eccentric phase can provide a novel stimulus and induce similar adaptations to those seen in the eccentrically-accentuated strength training protocols outlined above.

Despite the observed improvements in 10 m sprint performance for the H-L group, non-significant and unclear results were observed in COD performance for both groups, as assessed by the 505 test. It was hypothesized that the H-L group would significantly improve their performance during the 180° COD task and improve to a greater extent than the L-H group. This hypothesis was based on previous literature, which indicates that eccentrically-accentuated training can enhance the ability of athletes to produce and control force eccentrically, allowing for greater decelerations from faster approach velocities during COD tasks involving a large braking component [20,23,24]. Additionally, the greater braking impulses produced by athletes with superior eccentric strength capacities may also allow for more rapid reacceleration during a COD task as a result of the storage and utilization of elastic energy [1,24]. However, the results of the present study revealed that the plyometric interventions used were inadequate to produce significant improvements in COD ability of highly trained athletes despite the improvements in short distance sprint performance for the H-L group, indicating that an alternative stimulus may be required for COD enhancement. It has been suggested that the effectiveness of plyometric training for improving COD ability may be dependent on the manner in which they are performed, with regards to force-vector specificity [41,42,43,44,45,46]. Differences between plyometric training performed unilaterally compared to bilaterally have also been observed [19,47]. Brughelli and colleagues [10] noted traditional strength and power training exercises performed in a bilateral vertical manner have often failed to elicit improvements in COD performance, and exercises that more closely mimic the demands of COD tasks, including unilateral and bilateral horizontal and lateral jump training, more often report COD improvements. Some investigations into force-vector specificity and comparisons of unilateral and bilateral exercises within eccentric overload training have also been conducted, with unilateral multidirectional exercises producing more robust adaptations in COD performance, and bilateral vertical exercises displaying more robust adaptations in vertical jump, and sometimes linear sprint, performance [19,47]. Dello Iacono and colleagues [41] compared the effects of vertical and horizontal single-leg drop jump training in elite handball players and found stark differences in favor of the horizontal drop jump training group with regards to sprint and COD performance. It is therefore possible given the highly trained nature of the subjects that drop jumps performed in the present study may have been more suitable for enhancements in COD ability if they were performed in a unilateral multidirectional, rather than bilateral vertical, manner. Future research will be required to investigate the effects of eccentrically-accentuated plyometric exercises performed in a unilateral multidirectional manner, although the highly demanding nature of this type of exercise should be considered.

One limitation of the present study is the relatively small final sample, as this reduces the statistical power and ultimately limits the strength with which conclusions can be drawn based on the results. The power calculation that was done prior to beginning the study revealed that twelve subjects for each group would be sufficient to provide statistical power based on previous studies [12,15,18,22,33,38,44,45]. Thirty athletes were initially recruited, however, thirteen failed to complete the intervention. As this study was conducted to investigate hockey players specifically at the elite level, there was no opportunity to recruit additional participants in order to increase the sample size. It may have been useful to include some assessments of strength or analyses of kinetic and kinematic variables during the assessment of sprint and COD tasks in order to gain a greater understanding of adaptations to the training protocols and the mechanisms underlying any observed changes in performance. Nonetheless, some conjecture regarding these adaptations and mechanisms can be made based on the synthesis of these findings with previous research, and these considerations provide direction for future studies. To the best of the authors’ knowledge, no previous studies have examined the effects of a plyometric training intervention involving drop jumps performed in the manner of the H-L group in this study, with a greatly accentuated eccentric component. This is also one of very few studies that have investigated the effects of plyometric training in elite hockey players, and included both male and female athletes. Further, we believe the findings of this study are of strong practical relevance given the integration of the training protocol with the regular training program of the men’s and women’s national teams. These findings should, however, be interpreted with consideration of the specific population involved, and care should be taken when generalizing the results to athletic populations of different training status or sports with physical requirements that are different from hockey.

It should be noted that although the L-H group demonstrated no significant improvements in any performance variables based on analysis of group means, further analysis of individual responses reveals that two participants from this group responded poorly to the intervention and displayed a notably worsened performance in post-training sprint assessments (Figure 4, Figure 5 and Figure 6). It is estimated that with the exclusion of these two participants, comparable effects in sprint performance would likely have been observed between the groups following each training intervention. Although the exclusion of these data from the analysis is not statistically warranted, the effect of individual responses on overall group means in a relatively small sample should be considered when interpreting the results, and in particular when comparing the training protocols. This demonstrates the need for individualization of imposed training demands to elicit favorable adaptation in highly trained participants. As noted above, several previous studies have demonstrated drop jump training performed in the conventional manner can improve muscular power and sprint performance, and the weight of current literature still supports the use of conventional drop jumps as an effective method of training for power, speed, and perhaps COD [12,15,32,33,34].

## 5. Conclusions

This investigation aimed to compare the effects of two plyometric training protocols, involving either conventional drop jumps (L-H) or drop jumps performed with an accentuated eccentric phase (H-L), on sprint and COD performance in elite hockey players. Although the H-L training group significantly improved 10 m sprint time following the intervention, it is difficult to be sure that the intervention is truly responsible for this result. It is important to recognize that these are elite athletes and the duration of the test is very short and could be affected by variables including central and peripheral fatigue. This intervention was an addition to regular training and no statistically significant improvements were observed for any other performance variables. However, since speed and COD ability are integral to overall performance in sports such as hockey that require repeated rapid accelerations, decelerations, and changes of direction, the optimal methods and parameters of training for the enhancement of these athletic qualities are of constant interest to S&C practitioners. The findings highlight the practical difficulty in substantially enhancing speed and COD performance in highly trained elite athletes, particularly when the intervention is additional to large volumes of training being concurrently performed. However, the results also indicate promise for the use of eccentrically-accentuated plyometric training as a means of inducing novel stimuli for the enhancement of short distance sprint performance. It appears that whilst the drop jumps performed by the H-L group in this study were suitable to elicit some improvements in short distance sprint performance, enhancements in COD performance may require an alternative stimulus. Future research may include investigating the effects of eccentrically-accentuated plyometric training performed unilaterally and horizontally, rather than bilaterally and vertically. Additionally, the inclusion of strength assessments and analyses of kinetic and kinematic variables in training studies involving plyometric exercises performed with an accentuated eccentric phase would provide greater insight into the responses and adaptations following this type of training. Finally, it should be noted that, as discussed above, the weight of current literature still supports the use of drop jumps performed in the conventional manner as an effective means of enhancing muscular strength and power and related performance variables such as speed. The findings of this investigation present drop jumps performed in the manner of the H-L group as an alternative or additional approach to plyometric training.

## Figures and Tables

**Table 1:** Changes in performance from baseline to post-training for each group.

Performance Measure	High-to-Low (*n* = 9)			Low-to-High (*n* = 8)		
	Baseline	Post-Training		Baseline	Post-Training	
	Mean ± SD	Mean ± SD	ES	Mean ± SD	Mean ± SD	ES
10 m Sprint (s)	1.893 ± 0.08	1.851 ± 0.06 *	−0.44 (−0.75, −0.13)	1.868 ± 0.06	1.870 ± 0.04	0.05 (−0.37, 0.47)
20 m Sprint (s)	3.177 ± 0.14	3.155 ± 0.15	−0.07 (−0.37, 0.23)	3.141 ± 0.11	3.178 ± 0.09	0.34 (0.07, 0.61)
505 Time (s)	2.301 ± 0.07	2.293 ± 0.07	0.06 (−0.53, 0.65)	2.295 ± 0.09	2.291 ± 0.09	−0.03 (−0.43, 0.37)

* Significantly different from baseline, *p* < 0.05. ES: Cohen’s d effect size.
